# Comprehensive analysis reveals a 5-gene signature and immune cell infiltration in Alzheimer’s disease with qPCR validation

**DOI:** 10.3389/fgene.2022.913535

**Published:** 2022-08-25

**Authors:** Fanmao Jin, Yuemei Xi, De Xie, Qiang Wang

**Affiliations:** ^1^ Lishui People's Hospital, Lishui, Zhejiang, China; ^2^ School of Medicine, Xiamen University, Xiamen, Fujian, China; ^3^ Xiamen Key Laboratory of Translational Medicine for Nucleic Acid Metabolism and Regulation, Xiamen, Fujian, China

**Keywords:** Alzheimer’s disease, immune cell infiltration, neuroinflammation, bioinformatics, differentially expressed genes

## Abstract

Over 50 million people around the world currently are suffering from Alzheimer’s disease (AD) without any effective therapy. Neuroinflammation plays a pivotal role in AD, which leads us to probe the profile of immune cell infiltration in AD. Here, we analyzed a microarray dataset (GSE44770) containing 115 AD and 115 control samples to determine biomarkers and immune infiltration characteristics of AD by multiple bioinformatics methods. First, we identified 3,840 DEGs (1892 upregulated and 1948 downregulated) by using the limma package and 2,697 hub genes by constructing a weighted gene correlation network, and they had a total of 2,167 intersecting genes. Second, combining the LASSO logistic regression and SVM-RFE, we obtained five biomarkers (DGKG, MAP3K7IP2, NFKBIE, VIP, and PCCB), which may reveal the key pathogenetic features of AD and serve as diagnostic markers assessed by the ROC curve (AUC = 0.9716) and validation of another AD dataset (GSE33000) (AUC = 0.9388). Third, immune cell infiltration analysis revealed that compared with control samples, plasma cells, CD8 T cells, T follicular helper cells, and activated NK cells infiltrated less in AD; Monocytes, M2 macrophages, and neutrophils infiltrated more in AD. Neutrophils and activated NK cells demonstrated the most significant and negative correlation. Then, Spearman correlation analysis between the five biomarkers and immune infiltrating cells revealed that all of them were significantly associated with plasma cells. Finally, mRNA levels of VIP and PCCB were conformed in a murine AD model. In conclusion, DGKG, MAP3K7IP2, NFKBIE, VIP, and PCCB may be used as diagnostic markers of AD, and the disruption of the delicate immune balance may be a key process in the onset and development of AD.

## Introduction

Over 50 million people around the world currently suffer from Alzheimer’s disease (AD) (Chung et al., 2020). Yet to date, no effective therapy is available. Neuroinflammation plays a pivotal role in AD in addition to accumulation of extracellular β-amyloid (Aβ) and intracellular hyperphosphorylated tau (Heneka et al., 2015a). Combined multi-omics have uncovered more than 30 AD-risk loci, most of which are associated with microglia and innate immune responses, such as apolipoprotein E and triggering receptor expressed on myeloid cells 2 (Pimenova et al., 2018). This implies that neuroinflammation is not a bystander to the pathological process of AD but an essential contributor. This leads us to probe the profile of immune cell infiltration in AD.

Here, we determined five biomarkers and the immune infiltration characteristics of AD by multiple bioinformatics methods and found that in the brain tissues of AD patients, there is a tendency for the adaptive immune system to be activated and innate immunity to be suppressed, which may contribute to the pathological damage and cognitive impairment caused by immune dysregulation in AD.

### Data processing

GSE44770, an AD expression dataset, was downloaded from the Gene Expression Omnibus (GEO) database (https://www.ncbi.nlm.nih.gov/geo/) (Zhang et al., 2013). The data on autopsied brain tissues of the dorsolateral prefrontal cortex from 115 AD patients and 115 non-demented subjects were taken for the study. We sequentially normalized the gene expression data (normalizeBetweenArrays function), added missing values (impute.knn function), removed unannotated genes, discarded duplicates, and finally obtained the matrix for subsequent analysis, which includes 17,405 genes (exp_matrix_processed.txt).

### Recognition of differentially expressed genes

The limma package (Ritchie et al., 2015) in R was executed to identify DEGs in autopsied brain tissues of the dorsolateral prefrontal cortex between AD patients and non-demented subjects. *p* < 0.01 adjusted by the false discovery rate (FDR) and |log_2_ fold change (FC)| > 0.1 were considered as statistically significant.

### Weight gene correlation network analysis

The WGCNA package (Ritchie et al., 2015) in R was performed for WGCNA, with all genes included. First, the hclust function was used to inspect the hierarchical clustering traits of the sample. Second, the soft threshold power β was determined as 5 by the pickSoftThreshold function. The parameter TOMType was set as “unsigned”. The minimum module size should not be less than 30.

### Gene set enrichment analysis

GSEA is used to identify the differential expression of gene sets rather than individual genes (Subramanian et al., 2005). The clusterProfiler package in R (Subramanian et al., 2005; Yu et al., 2012) was used to conduct GSEA for screening Kyoto Encyclopedia of Genes and Genomes (KEGG) pathways and Gene Ontology (GO) terms that may be involved in AD of the GSE44770 dataset. All 17,405 genes and corresponding log_2_FC were included regardless of the *p*-value. The gene list can be found in Supplementary Table S1. *p* < 0.05 adjusted by FDR was considered statistically significant.

### Gene enrichment analysis

In total, 2,167 overlapping genes of DEGs and hub genes filtered from WGCNA were determined, which were considered the input gene set for GO and KEGG analysis. Biological processes (BP), cellular components (CC), and molecular functions (MF) comprise the GO terms. *p* < 0.05 adjusted by FDR was considered statistically significant.

### Scanning and validation of molecular markers

The least absolute shrinkage and selection operator (LASSO) regression algorithm is commonly performed on high-dimensional data and can accurately predict key features among thousands of variables (Tibshirani, 1996). Support vector machine-recursive feature elimination (SVM-RFE) is a feature selection algorithm widely used in genomics (Guyon et al., 2002; Kai-Bo et al., 2005), metabolomics (Mahadevan et al., 2008; Lin et al., 2012), and proteomics (Dao et al., 2017). The packages of glmnet and e1071 in R were used to implement algorithms of LASSO regression and SVM-RFE, respectively. Then, overlapping genes obtained by LASSO and SVE-RFE were taken as molecular markers of AD, which were validated with the receiver operating characteristic (ROC) curve.

### Immune cell infiltration

CIBERSORT, a method for cellular heterogeneity assessment, was used for the determination of immune cell infiltration (Newman et al., 2015). Samples with *p* < 0.05 were selected. In the present study, a total of 70 samples were retained for immune cell infiltration. Spearman correlation analysis was evaluated between recognized molecular markers and infiltrating immune cells.

### qPCR validation of gene expression in murine

The 5×FAD mice are a kind of canonical AD model mice (Oblak et al., 2021). All mice were kept at the Xiamen University Laboratory Animal Center with a 12-h light/12-h dark cycle and food and water *ad libitum*. All animal experimental procedures were approved by the Animal Care and Use Committee of Xiamen University. Total RNA in the brain cortex of 12-month-old WT and 5×FAD mice was extracted with the RNA extraction kit (#G3013, Servicebio, China); then, 2 mg of which was used for reverse transcription with the Evo M-MLV Mix Kit (#AG11728, Accurate Biology, China). The mRNA levels of relevant genes were determined by qPCR with SYBR Green Mix (#AH0104-B, SparkJade Biotechnology, China) and calculated. For statistical analysis, Student’s t-test was used. ns: not significant, *p < 0.05, **p < 0.01, and ***p < 0.001.

## Results

### Identification of DEGs in AD

**FIGURE 1:**
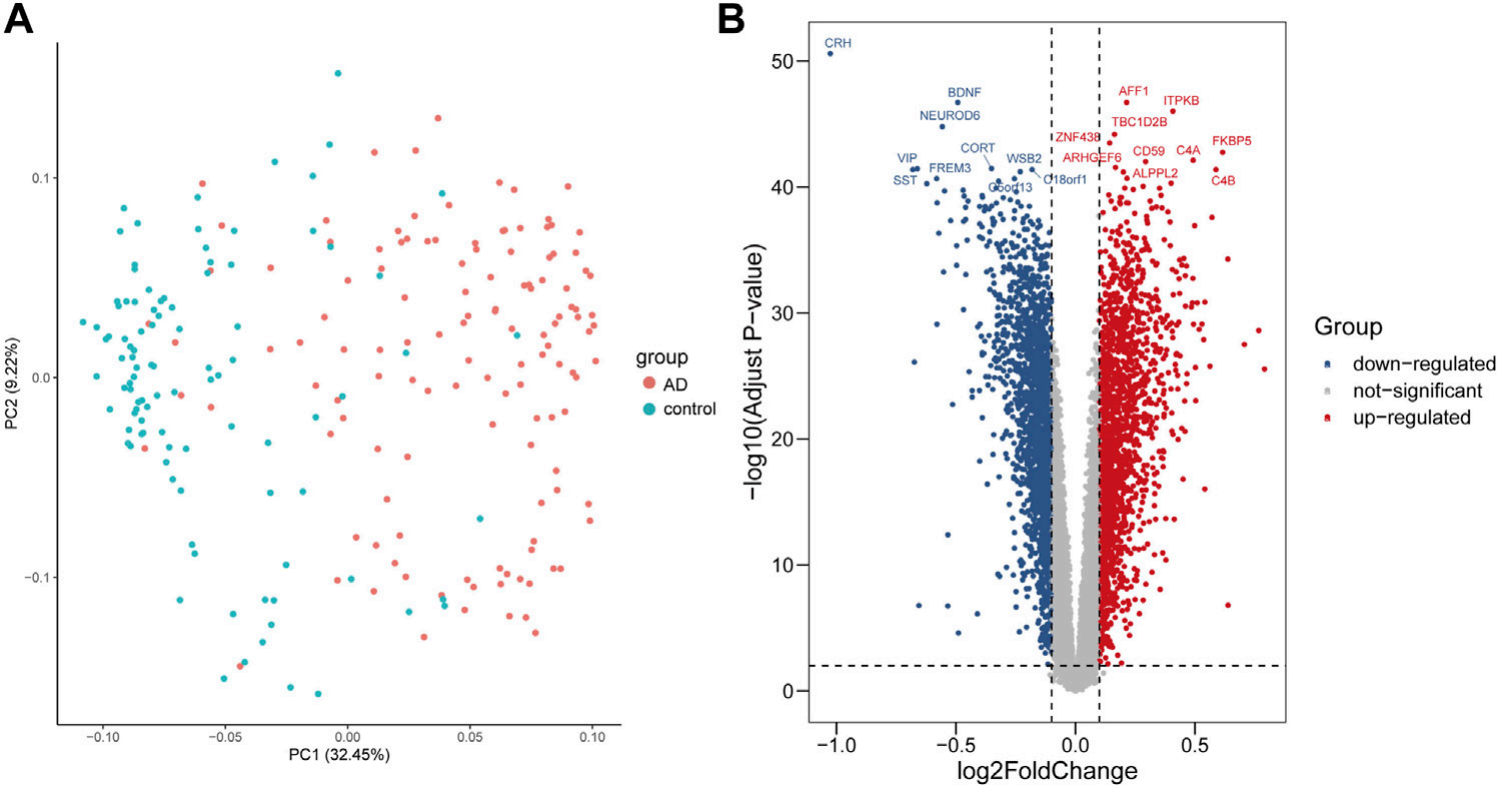
Visualization of the principal component analysis (PCA) and volcano plot of DEGs between AD and control samples. **(A)** PCA plot revealed that the two groups of AD and control clustered well after normalization. **(B)** Volcano plot showed DEGs, among which red dots represent upregulated genes, blue dots represent downregulated genes, and gray dots represent genes without significance.

The data from two groups clustered well after normalization indicated by principal component analysis (Figure 1A). Based on the given cut-off criteria, we identified 3,840 DEGs, of which 1892 were upregulated and 1948 were downregulated (Figure 1B, Supplementary Table S1, S2).

### Identification of the AD-related module by WGCNA

**FIGURE 2:**
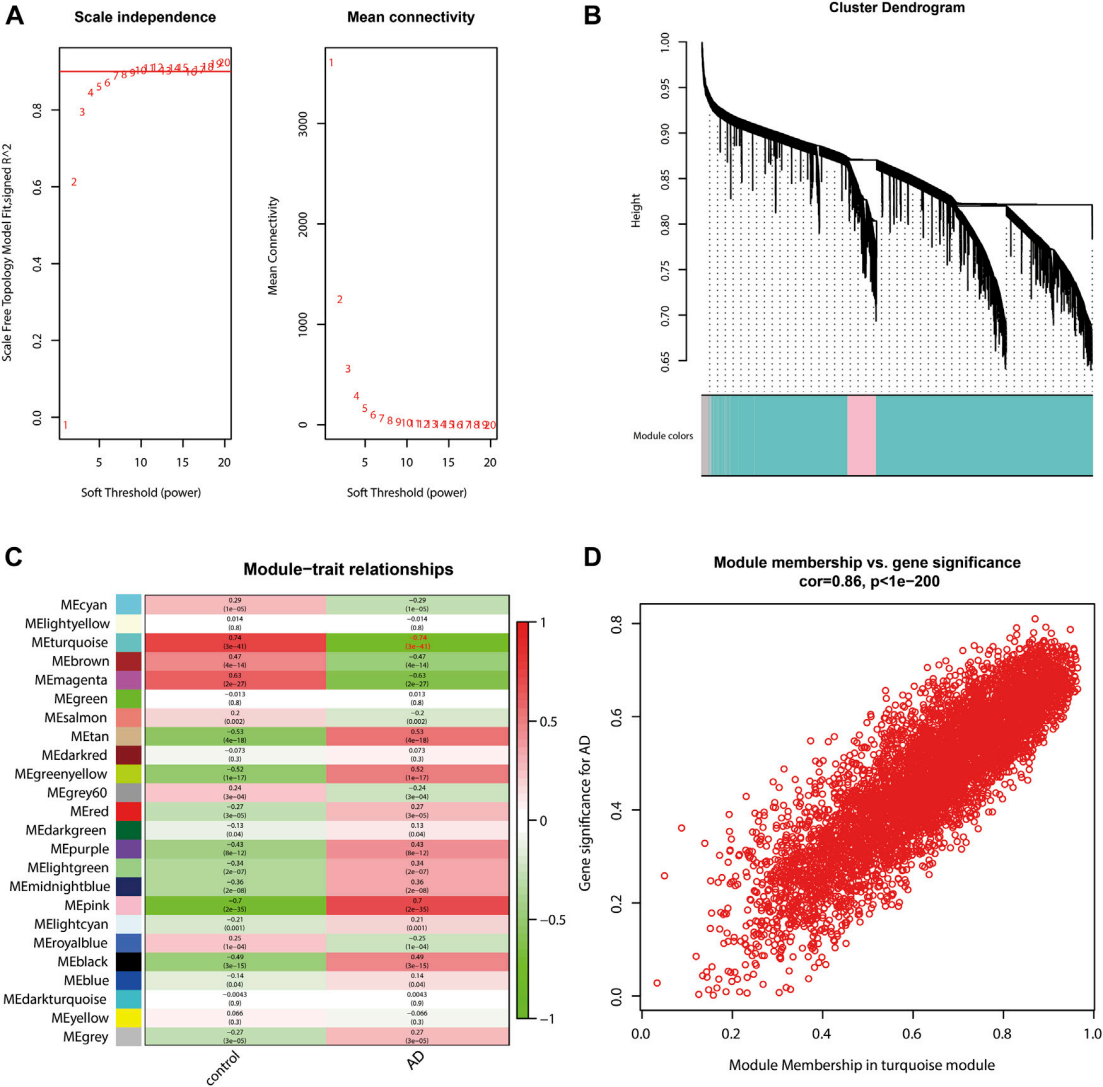
Identification of the AD-related module by WGCNA. **(A)** Soft threshold power β was set to 5. **(B)** Cluster dendrogram shows a total of 23 modules. Module size is as follows: black (668), blue (1,688), brown (1,497), cyan (96), darkgreen (45), darkred (49), darkturquoise (42), green (1,016), greenyellow (224), gray (1910), gray60 (77), lightcyan (83), lightgreen (72), lightyellow (57), magenta (459), midnightblue (88), pink (598), purple (331), red (799), royalblue (55), salmon (149), tan (167) turquoise (5,935), and yellow (1,300). **(C)** Correlation between modules and traits, among which the turquoise module is the most relevant to AD (correlation coefficient = −0.74; P = 3e-41). **(D)** Scatter plot shows the correlation between gene significance and module membership of individual genes in the turquoise module.

WGCNA was performed to identify the key module with the strongest relevance to AD. The soft threshold power β was set to 5 (Figure 2A). A total of 23 modules were determined (Figure 2B), with the turquoise module being the most relevant to AD (correlation coefficient = −0.74 and P = 3e-41; Figure 2C). The correlation between gene significance (GS) and module membership (MM) of individual genes in the turquoise module was presented as a scatter plot (Figure 2D). According to the cut-off criteria of |GS| > 0.5 and |MM| > 0.6, 2,697 genes in the turquoise module were determined as hub genes (Supplementary Table S3).

### Gene enrichment analysis

**FIGURE 3:**
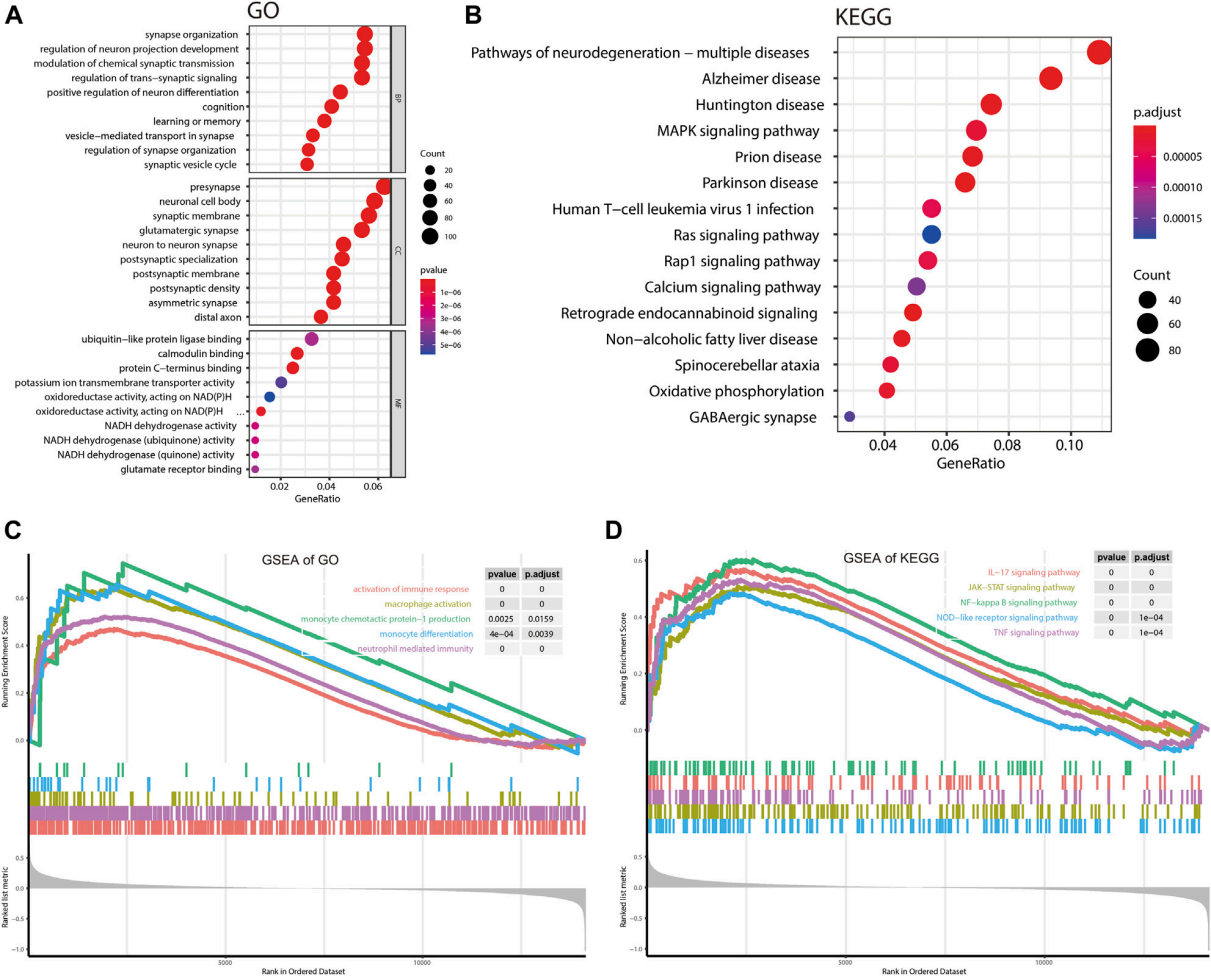
Gene enrichment analysis. **(A,B)** Bubble plot showing gene enrichment analysis of GO and KEGG. **(C,D)** GSEA for GO terms and KEGG pathways. BP, biological processes; CC, cellular components; MF, molecular functions.

There are 2,167 overlapping genes between DEGs and hub genes of the turquoise module (Supplementary Table S4), which were taken for further enrichment analysis of GO and KEGG (Figures 3A,B). GO analysis showed that genes were significantly enriched in synapse organization and regulation of neuro projection development, learning, or memory; KEGG analysis showed that genes were significantly enriched in Alzheimer’s disease and Huntington’s disease. All these results implied that AD is characterized by disordered homeostasis of the nervous system. As for GSEA, all genes and the corresponding log_2_FC (Supplementary Table S1) were included (Figures 3C,D). GSEA of GO showed that activation of the immune response, macrophage activation, and neutrophil-mediated immunity were significantly enriched, implying a role of immune response in AD. More specifically, GSEA of KEGG showed that inflammatory signaling pathways such as the IL-17 signaling pathway, JAK-STAT signaling pathway, and NF-kappa B signaling pathway were significantly enriched.

### Scanning and validation of molecular markers

**FIGURE 4:**
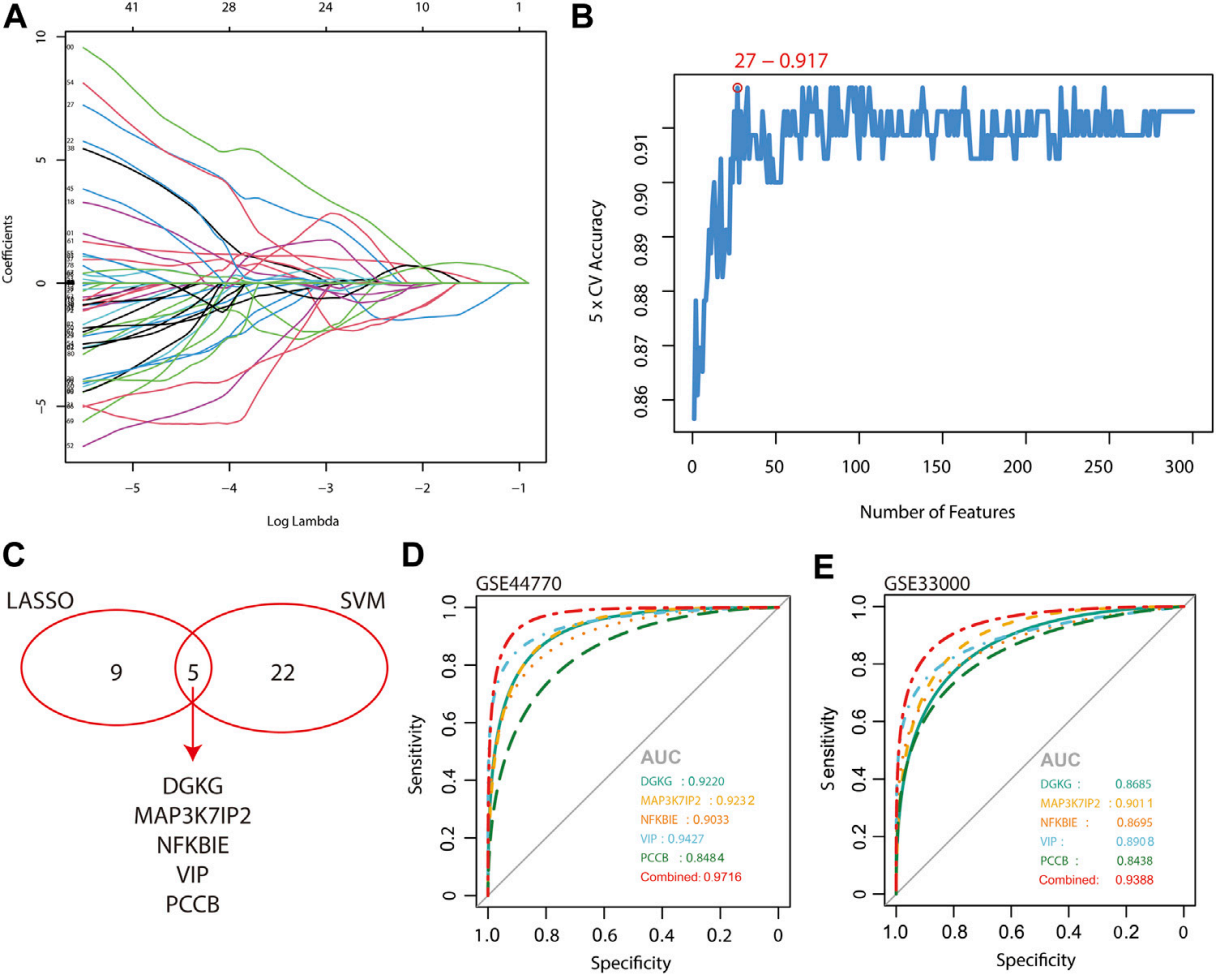
Scanning and validation of molecular markers by the algorithm of LASSO logistic regression and SVM-RFE. **(A)** Algorithm of LASSO logistic regression. **(B)** Algorithm of SVM-RFE. **(C)** Venn diagram shows shared biomarkers identified by LASSO logistic regression and SVM-RFE. **(D,E)** Diagnostic accuracy of the merged five biomarkers is shown by the ROC curve and validated in an external expression profile, GSE33000.

The 2,167 overlapping genes between DEGs and hub genes of the turquoise module were included for the algorithm of LASSO logistic regression and SVM-RFE. The samples are categorized into a training set and a test set in a ratio of 7:3 for LASSO regression, and as a result, 14 genes were identified (Figure 4A). The area under the curve (AUC) of the two sets implied a reliable accuracy of LASSO analysis and a favorable diagnostic capacity of the 14 genes by the ROC curve (AUC of the train set = 0.9798 and AUC of the test set = 0.9793; Supplementary Figure S1). A total of 27 genes were determined by the SVM-RFE algorithm with 5-fold cross validated accuracy (Figure 4B). Finally, we identified five genes shared by the two algorithms, namely, DGKG, MAP3K7IP2, NFKBIE, VIP, and PCCB (Figure 4C), and the ROC curve indicated high diagnostic accuracy of the merged five genes with reasonableness and appropriateness of combining LASSO regression and SVM-RFE (combined AUC = 0.9716; Figure 4D). Further validation was performed in another expression profile of AD, GSE33000 (Narayanan et al., 2014), and the result implied high sensitivity and specificity of DGKG, MAP3K7IP2, NFKBIE, VIP, and PCCB for diagnosing Alzheimer’s disease (combined AUC = 0.9388; Figure 4E).

### Assessment of immune cell infiltration

**FIGURE 5:**
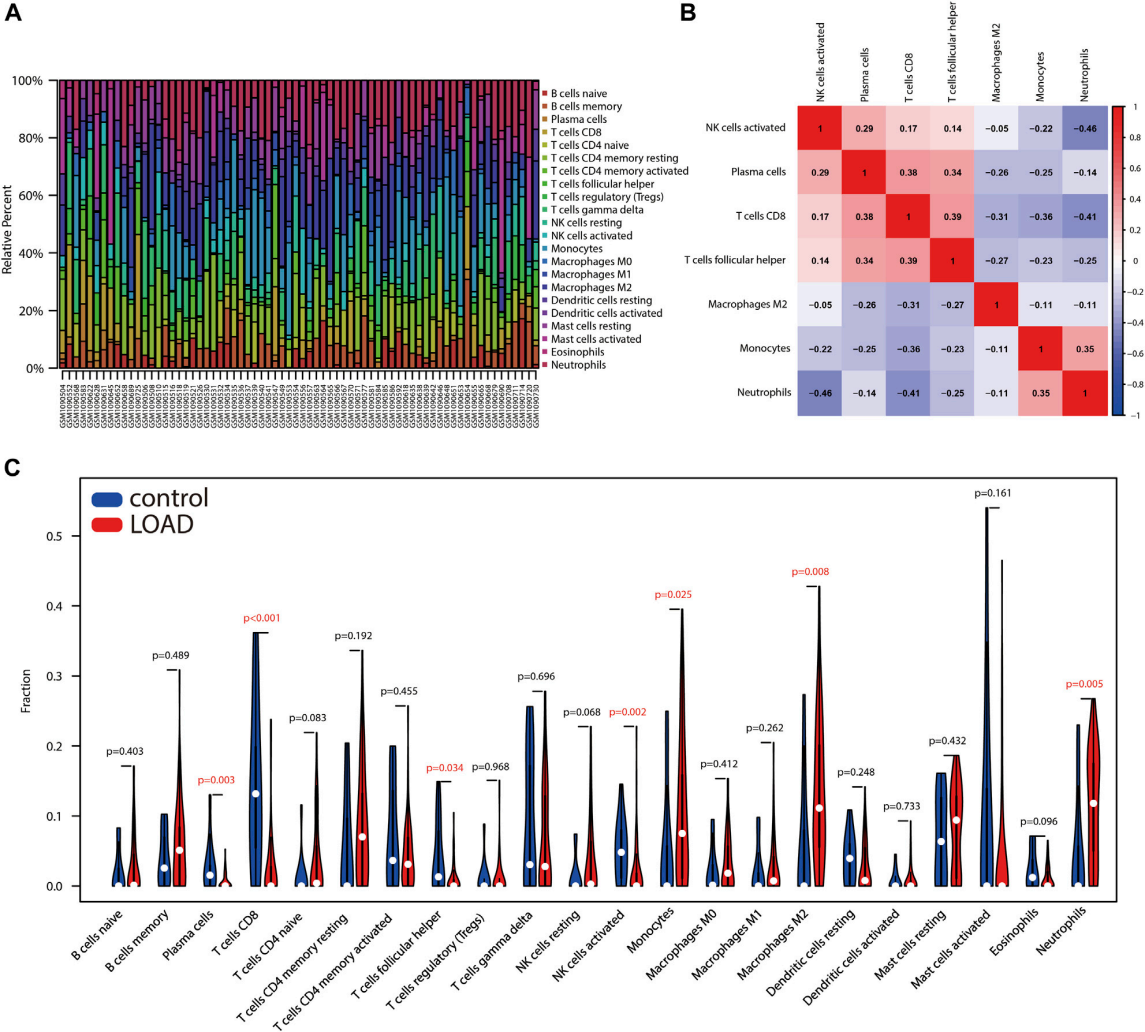
Assessment of immune cell infiltration between AD and control samples. **(A)** Box plot shows the relative proportion of different types of immune cells in samples. **(B)** Heatmap indicates the correlation of different types of immune cells with significant differences. **(C)** Violin plot shows differences in immune infiltrating cells between AD and control samples.

Immune cell infiltration traits were assessed by CIBERSORT, followed by visualization. Figure 5A showed the immunocyte composition of each sample. A total of seven types of immune cell infiltration significantly differed between control and AD samples, and their correlation was evaluated (Figures 5B,C). Specifically, compared with control samples, plasma cells, CD8 T cells, T follicular helper cells, and activated NK cells infiltrated less in AD; monocytes, M2 macrophages, and neutrophils infiltrated more in AD (Figure 5C). The degree of correlation between different immune cells implies their potential inter-regulatory link. Among them, neutrophils and activated NK cells demonstrated the most significant and negative correlation, and T follicular helper cells indicated the most significant and positive correlation with CD8 T cells (Figure 5B).

### Correlation between the 5-gene signature and infiltrating immune cells

**FIGURE 6:**
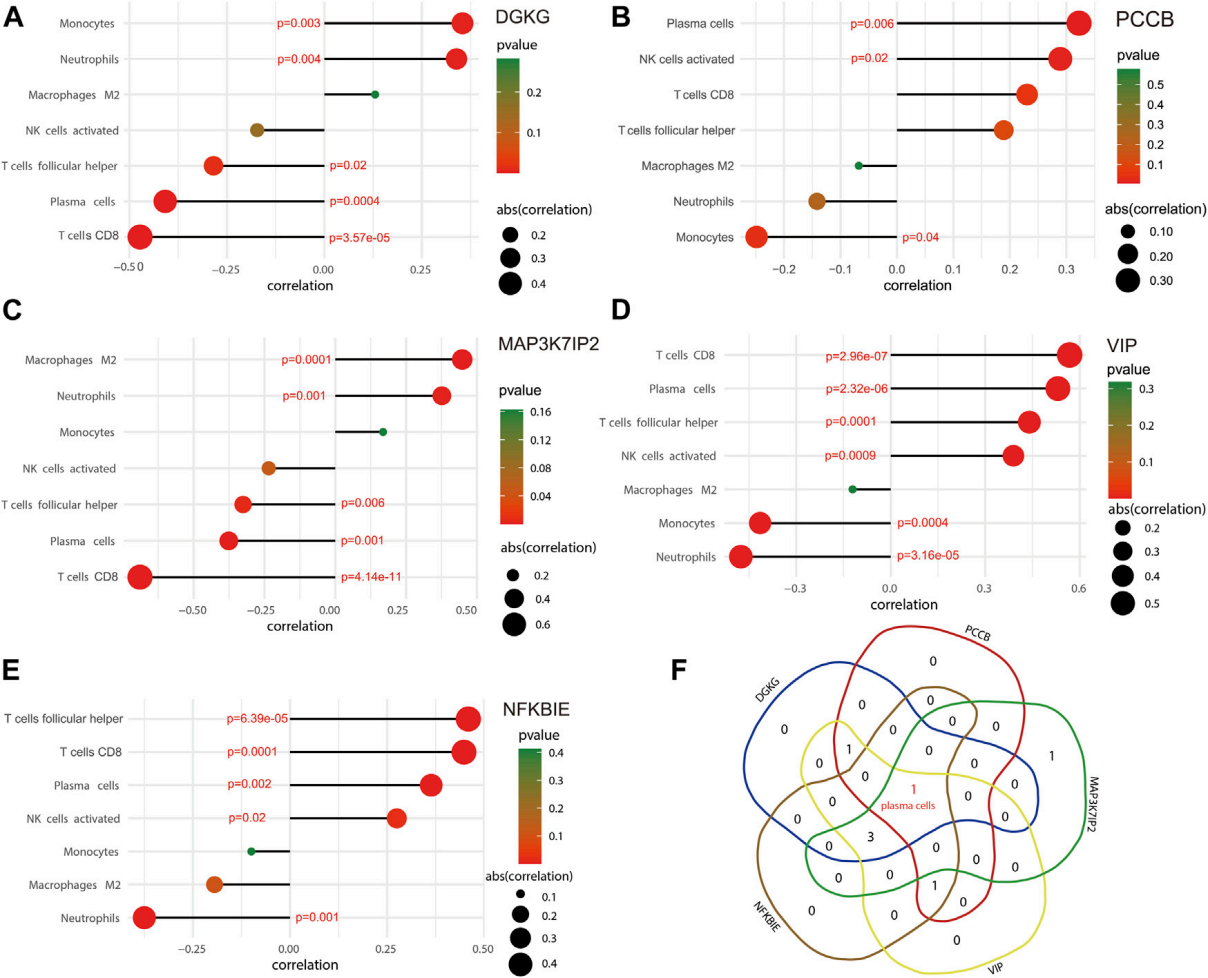
Correlation between the 5-gene signature and infiltrating immune cells. **(A)** Immune infiltrating cells significantly correlated with DGKG. **(B)** Immune infiltrating cells significantly correlated with PCCB. **(C)** Immune infiltrating cells significantly correlated with MAP3K7IP2. **(D)** Immune infiltrating cells significantly correlated with VIP. **(E)** Immune infiltrating cells significantly correlated with NFKBIE. **(F)** Venn diagram indicates that plasma cells is the only term of immune infiltrating cells that significantly correlates with all the five biomarkers.

The ggstatsplot package was used for Spearman correlation analysis between the 5-gene signature and infiltrating immune cells. The results showed that DGKG positively correlated with monocytes (correlation = 0.354; *p* = 0.003) and neutrophils (correlation = 0.339; *p* = 0.004), and negatively correlated with T follicular helper cells (correlation = −0.285; *p* = 0.02) and plasma cells (correlation = −0.408, *p* = 0.0004), together with CD8 T cells (correlation = −0.473; *p* = 3.57e-05) (Figure 6A); PCCB positively correlated with plasma cells (correlation = 0.322; *p* = 0.006) and activated NK cells (correlation = 0.289; *p* = 0.02) and negatively correlated with monocytes (correlation = −0.249, *p* = 0.04) (Figure 6B). Similarly, there were several infiltrating immune cells correlated with MAP3K7IP2, VIP, and NFKBIE (Figures 6C–E). The term of plasma cells is the only immune infiltrating cells that significantly correlate with all genes in the 5-gene signature (Figure 6F). These results may reveal a potential link between the 5-gene signature and traits of infiltrating immune cells, as well as regulatory effects of the 5-gene signature on infiltrating immune cells. The combined effects of the 5-gene signature may be a driving factor in regulating infiltrating immune cells.

### qPCR validation of the 5-gene signature in murine

**FIGURE 7:**
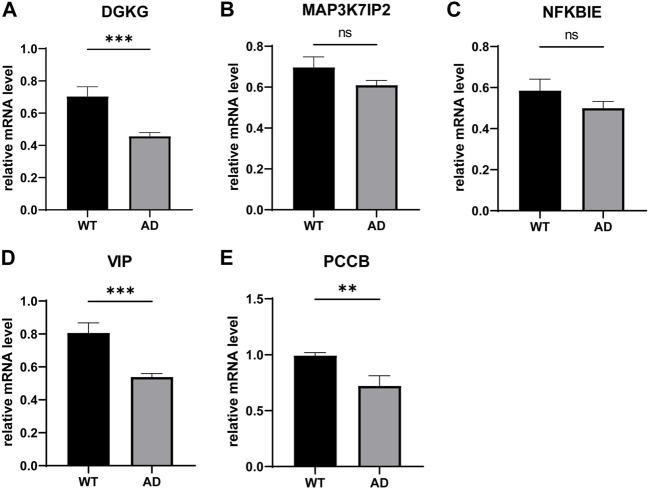
qPCR validation of the 5-gene signature in murine. ns: not significant, *p < 0.05, **p < 0.01, and ***p < 0.001.

We determined the mRNA levels of these five genes using qPCR and found that VIP and PCCB expressions were downregulated compared to the control group, which is consistent with our previous analysis. However, the DGKG expression profile was contradictory to our aforementioned results; furthermore, we found that the expression levels of MAP3K7IP2 and NFKBIE in the AD group were not significantly different from those in the WT group (Figures 7A–E).

## Discussion

AD comprises the predominant type of dementia without any effective therapy. In-depth exploration of the molecular mechanisms of AD can help identify new therapeutic targets. The role of neuroinflammation in AD has been extensively examined over recent years (Heneka et al., 2015a; Calsolaro and Edison, 2016; Ryu et al., 2018), yet its exact contribution remains unclear, which guided us to explore the immune infiltration characteristics of AD.

Here, we applied comprehensive bioinformatics methods to explore new mechanisms of AD. In total, 2,167 genes were identified by combining DEG and WGCNA, which were mainly enriched in pathways involving disordered homeostasis of the nervous system. GSEA of GO and KEGG implied a pivotal role of neuroinflammation in AD, such as the IL-17 signaling pathway, JAK-STAT signaling pathway, or NOD-like receptor signaling pathway.

Then, a 5-gene signature (DGKG, MAP3K7IP2, PCCB, VIP, and NFKBIE) was identified by combining the LASSO logistic regression and SVM-RFE, two machine learning methods searching for the best variable. Briefly, DGKG (diacylglycerol kinase gamma), exclusively expressed in the cerebellum, modifies protein kinase C gamma to regulate cerebellar motor coordination. Considering the potentially critical role of the cerebellum in cognitive impairment of AD, how DGKG participates in AD warrants further research (Jacobs et al., 2018). MAP3K7IP2 (mitogen-activated protein kinase 7-interacting protein 2), an activator of TAK1, is necessary for the IL-1-induced activation of NF-κB and JNK(Hu et al., 2014). PCCB (propionyl-CoA carboxylase subunit beta) is a mitochondrial enzyme involved in lipid and amino acid metabolism (Kalousek et al., 1980; Jiang et al., 2005; Chasman et al., 2009). The aberrant methylation of PCCB is linked to autism spectrum disorder by causing mitochondrial dysfunction (Stathopoulos et al., 2020). VIP (vasoactive intestinal peptide) has been shown to be neuroprotective and plays an essential role in the acquisition of learning and memory (Gozes et al., 1996). NFKBIE (NFKB inhibitor epsilon) can set NF-κB in an inactive state by cytoplasmic retention of REL proteins (Whiteside et al., 1997; Hoffmann et al., 2002). Downregulation of NFKBIE may lead to aberrant upregulation of NF-κB, thus participating in the development of AD. We further validated the mRNA levels of these genes by qPCR in an AD mouse model and found that VIP, PCCB, and DGKG were down-regulated compared to the control group, while MAP3K7IP2 and NFKBIE in the AD group were not significantly different from those in the WT group. *In vivo* and *in vitro* result conflicts might be due to species differences. Collectively, the signature may reveal the key pathogenetic features of AD, which need further research.

To further investigate the role of immune cell infiltration in AD, we performed a comprehensive evaluation of AD immune infiltration using CIBERSORT. We found less infiltration of plasma cells, CD8T cells, T follicular helper cells, and activated NK cells and more infiltration of monocytes, M2 macrophages, and neutrophils in AD. Intriguingly, the former is all classified as adaptive immunity, and the latter is all classified as innate immunity; this implies a tendency of the activated adaptive immune system and suppressed innate immunity, which is consistent with the literature and may contribute to the pathological damage and cognitive impairment caused by immune dysregulation in AD. Specifically, an AD transgenic mouse model lacking adaptive immune populations (T cells, B cells, and natural killer cells) showed higher Aβ deposition and exacerbated neuroinflammation (Marsh et al., 2016). Preclinical and epidemiological studies have shown that the activated innate immunity is a key factor in promoting the development of AD (Heneka et al., 2015b). Therefore, appropriate suppression of the activated adaptive immunity and restoration of the suppressed innate immunity may be a strategy for AD treatment.

There are several limitations to the present study. First, sample size is inadequate; second, AD model mice instead of human-derived tissues were used for mRNA expression validation of the screened genes. Overall, further basic and clinical research studies are still warranted to conform our findings.

## Conclusion

In conclusion, activated adaptive immunity and suppressed innate immunity may act as underlying causes of AD, and appropriate suppression of the activated adaptive immunity and restoration of the suppressed innate immunity may be a therapeutic strategy for AD treatment. DGKG, MAP3K7IP2, NFKBIE, VIP, and PCCB may play a pivotal role in AD and can be used as diagnostic markers and therapeutic targets of AD.

## Data Availability

The datasets presented in this study can be found in online repositories. The names of the repository/repositories and accession number(s) can be found in the article/Supplementary Materials.
