# Supplementary figures and images for: Comprehensive analysis reveals a 5-gene signature and immune cell infiltration in Alzheimer’s disease with qPCR validation
Source: Front Genet. 2022 Aug 25;13:913535. doi: 10.3389/fgene.2022.913535 (PMC9454400; doi:10.3389/fgene.2022.913535)

GSE44770

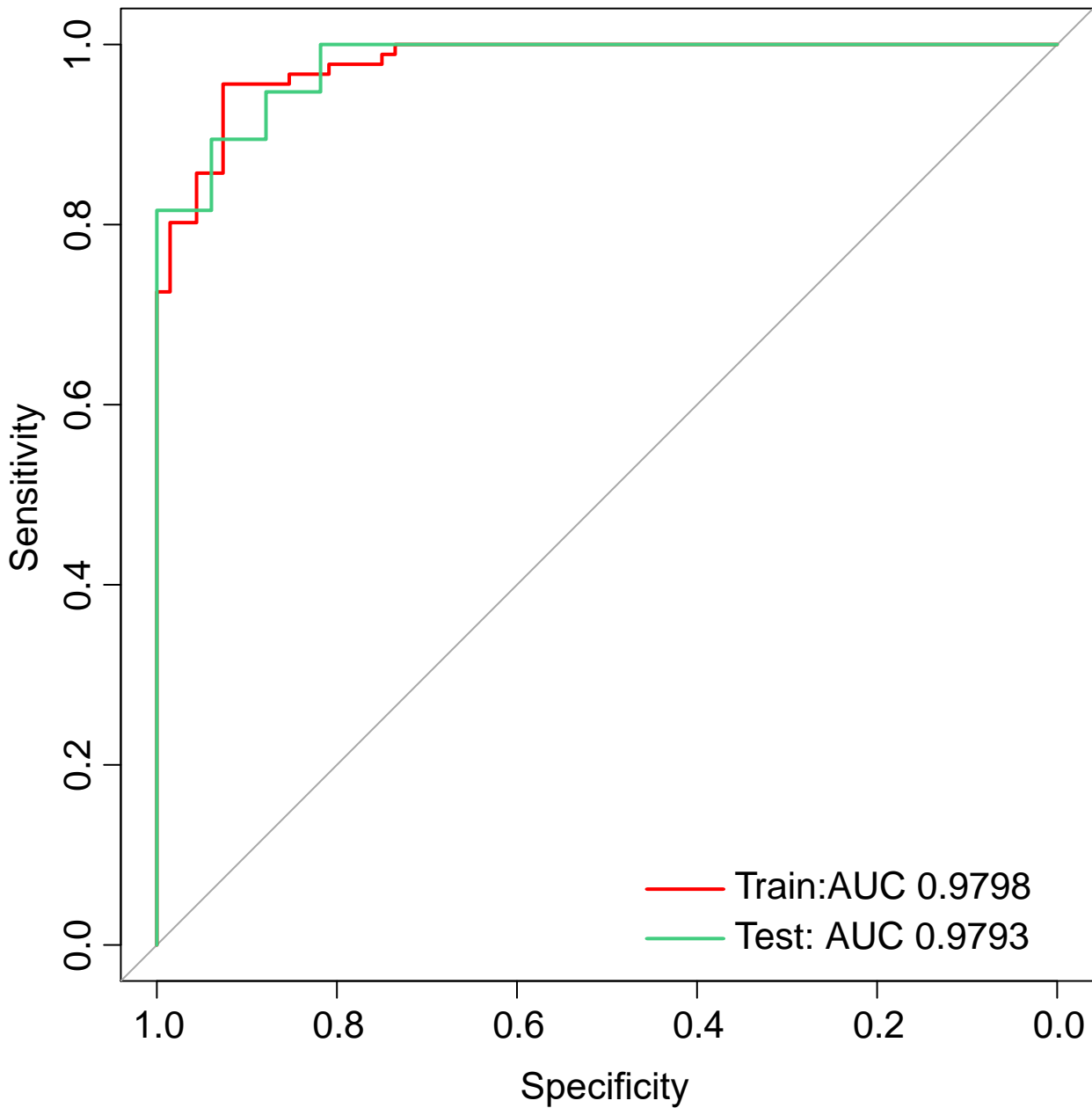

Supplement: Supplementary file 1 [file Image1.pdf]
